# Metal-free supercapacitor with aqueous electrolyte and low-cost carbon materials

**DOI:** 10.1038/srep39836

**Published:** 2017-01-05

**Authors:** Nicklas Blomquist, Thomas Wells, Britta Andres, Joakim Bäckström, Sven Forsberg, Håkan Olin

**Affiliations:** 1 Mid Sweden University, Department of Natural Sciences, Sundsvall, SE-851 70, Sweden; 2 STT Emtec AB, Sundsvall, SE-852 29, Sweden

## Abstract

Electric double-layer capacitors (EDLCs) or supercapacitors (SCs) are fast energy storage devices with high pulse efficiency and superior cyclability, which makes them useful in various applications including electronics, vehicles and grids. Aqueous SCs are considered to be more environmentally friendly than those based on organic electrolytes. Because of the corrosive nature of the aqueous environment, however, expensive electrochemically stable materials are needed for the current collectors and electrodes in aqueous SCs. This results in high costs for a given energy-storage capacity. To address this, we developed a novel low-cost aqueous SC using graphite foil as the current collector and a mix of graphene, nanographite, simple water-purification carbons and nanocellulose as electrodes. The electrodes were coated directly onto the graphite foil by using casting frames and the SCs were assembled in a pouch cell design. With this approach, we achieved a material cost reduction of greater than 90% while maintaining approximately one-half of the specific capacitance of a commercial unit, thus demonstrating that the proposed SC can be an environmentally friendly, low-cost alternative to conventional SCs.

The number of publications regarding electric double-layer capacitors (EDLCs) or supercapacitors (SCs) and related applications is rapidly increasing. Because of the excellent performance of SCs in handling short peak power pulses with high efficiency and their long lifetime and superior cyclability, their applications range from small consumer electronics to electric vehicles and stationary grid applications^1^,^2^,^3^,^4^,^5^. In stationary applications, an SC is used to provide power stabilization by handling short power surges in the grid or as a buffer to compensate for the irregular supply of generated electricity from solar cells and windmills^2^. In automotive applications, an SC can enhance battery life, enhance the efficiency of regenerative braking or function in combination with fuel cells to handle peak power demands^3^,^4^,^5^. However, the high cost of SCs is a substantial issue for large-scale commercial use, thus leading to a need for environmentally safe, low-cost materials and simplified manufacturing processes^1^,^2^,^6^,^7^.

Most commercial SCs use organic electrolytes and highly porous carbon electrodes coated onto aluminum foil^1^,^6^. The main advantage of organic electrolytes is their wide electrochemical stability window (approximately 2.7 V); however, compared with aqueous alternatives, they are, expensive, flammable and, in some cases, toxic. Although aqueous electrolytes have a narrower electrochemical stability window (approximately 1.23 V), they are nonflammable, inexpensive, have higher ion conductivity and give often rise to higher capacitance due to smaller ions^1^,^6^,^8^.

The favorable cost and environmental aspects of SCs with aqueous electrolytes are promising; however, the development of low-cost current collectors for such SCs poses a substantial challenge^6^,^8^,^9^. The aggressive nature of the aqueous environment demands electrochemically stable materials in both the electrode and current collector to prevent oxidation leading to high interfacial resistance^9^. Gheytani *et al*.^9^ have used chromate-conversion-coated aluminum to avoid corrosion of aluminum current collectors in aqueous lithium-ion batteries. With this technique, they have maintained the current collectors’ low cost compared with that of well-known corrosion-resistant materials such as titanium, stainless steel, nickel and platinum. Béguin *et al*.^6^ have reported that stainless steel is most suitable for neutral (pH 7) aqueous electrolytes and that the use of acidic solutions (low pH) severely restricts commercialization because of the prohibitive price of corrosion-resistant current collectors (e.g., gold or platinum).

The four connected problems in designing environmentally friendly and cost-effective SCs may thus be summarized as: 1) electrolyte, 2) electrode, 3) current collector and 4) the interface between the electrode and current collector.

Here, we report the design of an SC based on an aqueous electrolyte and a mix of nanographite, activated carbon (AC) and nanofibrillated cellulose (NFC) as the electrode material. We have taken an alternative approach to common current collectors, moving away from metal and instead using graphite foil to avoid current-collector corrosion and to exploit the potentially low resistance at the carbon-carbon interface. In this paper, we describe the processing of a potentially low-cost and metal-free SC with an aqueous electrolyte and analyze the device’s performance in terms of capacitance and series resistance.

## Results and Discussion

### Electrode coating

To investigate the influence of various mixing ratios between nanographite and AC in terms of capacitance and the SC series resistance (ESR) in the SCs, 5 different samples (A–E) were prepared. We coated samples A to E onto graphite foil by pouring the sample dispersions into frames, leveling them with a coating blade to a 2 mm wet height and then drying them at room temperature. The sample composition are shown in Table 1, in which the active material composition describes the ratio between the nanographite and AC. All samples had an addition of 10% NFC as binder.

**Figure 1:**
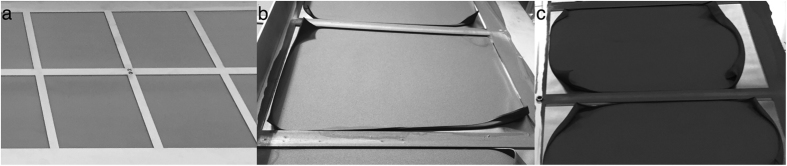
(**a**) Sample A coated onto graphite foil. The only visible difference among samples A, B and C was a slight difference in color, in which samples B and C were a slightly darker shade of gray. (**b**) Sample D coated onto graphite foil with some shrinkage and deformation effects at the edges of the electrode. (**c**) Sample E coated onto graphite foil with large shrinkage and deformation at the edges and along the sides of the electrode.

Samples A, B and C showed good adhesion to the graphite foil and resulted in smooth electrodes; see Fig. 1a. Samples D and E showed less adhesion, large shrinkage and deformation during drying. These effects were more pronounced in sample E than in sample D and thus appear to be a result of the greater proportion of AC in sample E. Sample E was also very brittle and fell apart when the electrodes were disassembled from the frames. Figure 1b and c shows images of samples D and E coated onto graphite foil, respectively.

The large shrinkage was most probably due to the particle size and shape in the electrode. The nanographite consists of micrometer-sized and nanometer-thin graphene-like flakes^10^, thus yielding a robust, smooth and fairly flexible electrode when combined with the NFC binder. The ACs are micrometer-sized clusters of irregularly shaped porous carbon particles. in cases in which the amount of ACs was dominant, the shrinkage increased and the electrode became more brittle. These effects are probably a result of the change in size and shape distribution of the particles in the electrode, thereby affecting the interaction to the binder and the latching structure from the nanographite flakes.

**Figure 2:**
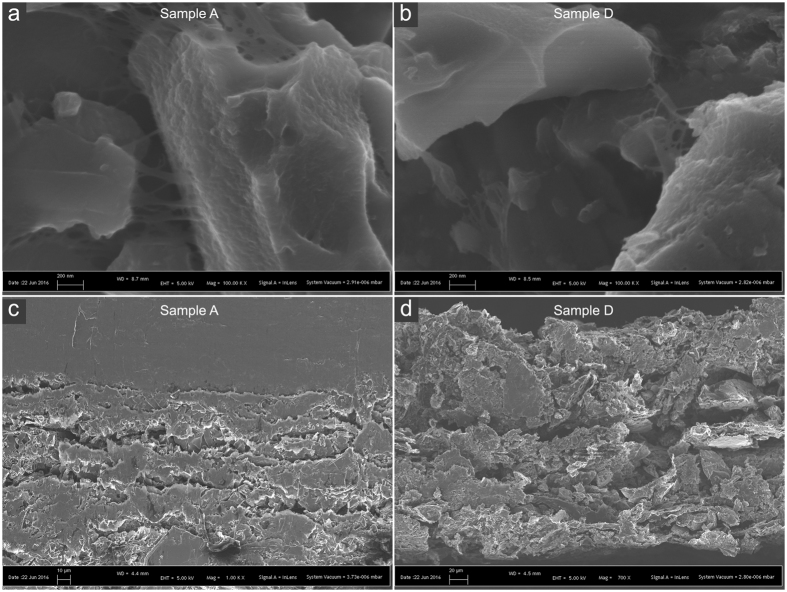
Surface image on sample A (**a**) and sample D (**b**) showing the web-like nanofibrillated cellulose (NFC) structure between particles of nanographite and AC. (**c**) and (**d**) Show cross-section images of sample A and D respectively.

Figure 2 shows SEM-images of the electrode coated graphite foil from sample A and D. From the surface images (a) and (b) it can clearly be seen that the NFC forms a web-like structure holding the nanographite and ACs together. Furthermore, it can be assumed from the same images that NFC also formed a film covering the AC particle (to the right in the images) since they got a cloud-like surface with soft edges, which are not visible on the nanographite particles (to the left in the images). The cross sectional images show that both sample A (c) and sample D (d) has relatively large cavities, or gaps between clusters of particles, which could be reduced if the electrodes were compressed. The solid gray area in the top of (c) is the graphite foil.

### Electrical measurements

**Figure 3:**
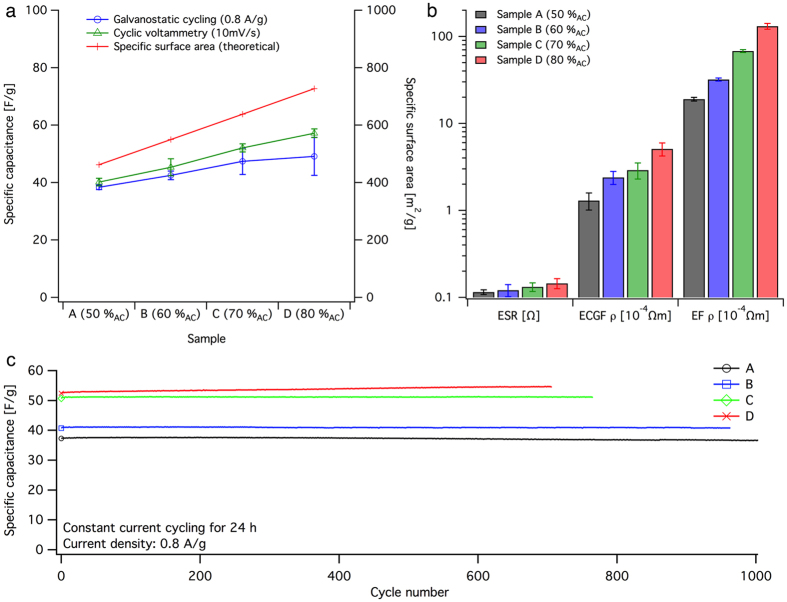
(**a**) Data plot of measured specific capacitance on SC units from samples A to D from both galvanostatic cycling (blue, ○) and cyclic voltammetry (green, △). The red plot (+) corresponds to the theoretical specific surface area of the electrode on the basis of the material composition. (**b**) Bar plot of SC series resistance (ESR), the electrical resistivity in the electrode-coated graphite foil (ECGF *ρ*) and the electrical resistivity in the electrode film (EF *ρ*) for samples A to D. The value axis has a logarithmic scale. (**c**) Data plot from 24 h constant current cycling (0 V − 1 V − 0 V) on SC units from samples A to D.

Figure 3a shows data plots of the measured specific capacitance and theoretical electrode surface area for SC units from samples A to D coated on graphite foil. All units had a symmetrical set of electrodes (equal mass loading). Notably, sample E could not be evaluated on graphite foil because of excessive shrinkage and deformation during drying, which resulted in cracks and insufficient adhesion.

The discharge rates between galvanostatic cycling (GC) and cyclic voltammetry (CV) measurements differed in this setup. The discharge rate was approximately twice as high during GC compared with that during CV, thus resulting in a shorter time for ion diffusion and explaining the lower specific capacitance values measured by GC. At low discharge rates, the ions have sufficient time to diffuse into the deep pores of the electrode, whereas at high rates, only the large, easily accessible pores are accessed^6^,^11^,^12^. This difference is more evident in the case of the electrodes with a larger proportion of AC because they have a substantially higher theoretical specific surface area. The difference between the measured and theoretical capacitance stemming from the increases amount of ACs may be attributed to insufficient electrolyte wetting or unavailable surface area. The NFC binder may form a thin film covering part of the available pores, essentially blocking them. This can be assumed from the SEM images in Fig. 2a,b. Another scenario is an uneven particle distribution in the electrode with clusters of poorly connected ACs contributing with an inaccessible surface area.

Figure 3b shows a bar plot of the ESR of the unit, the electrical resistivity in the electrode-coated graphite foil (ECGF *ρ*) and the electrical resistivity in the electrode film (EF *ρ*) for samples A to D. The electrical resistivity was derived from sheet resistance. The graphite foil was 200 *μ*m thick, and the electrodes from samples A to D were between 200 *μ*m and 250 *μ*m thick. The measurements of electrical resistivity clearly showed that the interfacial resistance between the graphite foil and the electrode was low. The electric resistivity in the electrode-coated graphite foil was more than one order of magnitude lower than that in the electrode film. Despite the large difference in electrical conductivity in the electrodes and the fact that the units were evaluated without applied pressure (uncompressed), the ESR was fairly low for all samples, thus indicating that the graphite foil is a good candidate as a low-cost current collector in aqueous environment. The ESR was also low in comparison to some other aqueous unconventional SCs^13^,^14^, but still large for high power applications.

Xiaohang Z. *et al*.^15^ reported on a 20 V stack of aqueous SCs with titanium plates as current collectors and CNT-polypyrrole electrodes; showing an ESR (compressed stack) of 16 mΩ per cell, which is low compared to the graphite foil at approximately 100 mΩ. Notably, the electrode coated graphite foil SC units were uncompressed and coated at room temperature without any sintering or calendering. The ESR could potentially be further decreased by vacuum-drying the electrodes at elevated temperature with subsequent calendering, to reduce the large cavities shown in Fig. 2c,d and enhance the contact between particles in the electrode. Another interesting feature of the metal foil, such as Ti, is as bipolar plate between cells in a stack^15^, this is unfortunately not possible with graphite foil since its slightly porous structure do not prevent ion transport through the foil.

Figure 3c shows cycle stability data from 24 h constant current cycling (0 V − 1 V − 0 V) at a current density of 0.8 A/g (2.5 mA/cm^2^) for SC units A to D. No change in specific capacitance could be observed for unit A, B and C during 24 hours cycling, but a small improvement was observed for SC unit D. Unit D showed approximately 4% higher specific capacitance after 24 h (706 cycles), compared to cycle 2. This can be a consequence of insufficient time for electrolyte wetting during SC unit assembling, resulting in continued wetting of deeper pores during cycling. The cyclability study indicates good cycle stability, despite the simple electrode manufacturing by coating with water-based binder solution together with the use of aqueous electrolyte in the SC units. An explanation for this could be that high shear forces were used both for nanographite exfoliation and during addition of NFC and AC. When dried, the particles in the dispersion are held together by the nano-fibrils in a web-like formation forming a robust composite with good mechanical stability and wet strength^16^.

**Figure 4:**
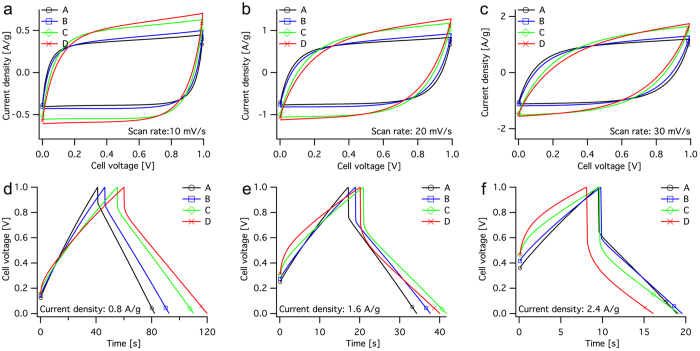
Shows I–V curves from cyclic voltammetry measurements with different scan rates in (**a**–**c**) and constant current curves from galvanostatic cycling with different current densities in (**d**–**f**). In CV, the SC units A (black, ○), B (blue, □), C (green, ◇) and D (red, x) were cycled with the scan rates (**a**) 10 mV/s, (**b**) 20 mV/s and (**c**) 30 mV/s. In GC the same units were cycled between 0 V and 1 V, and the charge and discharge current densities were (**a**) 0.8 A/g (2.5 mA/cm^2^), (**b**) 1.6 A/g (5 mA/cm^2^) and (**c**) 2.4 A/g (7.5 mA/cm^2^).

Figure 4a–c shows I–V curves from CV measurements of SC units A to D with three different scan rates, 10 mV/s (a), 20 mV/s (b) and 30 mV/s (c). The shapes of the CV measurements showed that no reactions occurred, other than electrostatic charging and discharging. The differences among the units were evident the curve shapes and the current plateaus, which showed higher capacitance (plot area) for units with grater amounts of activated carbons. This was most pronounced at a scan rate of 10 mV/s, at higher scan rates the curvature shifted substantially for unit C and D. The different curve shapes further indicated that, with increasing amount of AC, a higher cell voltage is required to obtain the same charge current density as that of unit A (the bending distance is longer for units B to D), thus indicating greater resistance to charge transfer (charge propagation) in the electrode. This is most evident at scan rates of 20 mV/s and 30 mV/s and might be a result of a higher electrode resistance combined with a higher surface area, thus leading to longer diffusion times for the ions. In addition, the current plateaus at 10 mV/s were flat or slightly bent during discharging but steeper during charging. This effect was more apparent in the voltammograms of units with grater amounts of AC and may be a direct result of unit leakage current. The leakage current was generated from the internal resistance and thus increased with increasing cell voltage, resulting in a measured charge current greater than the measured discharge current.

Figure 4d–f shows constant-current curves from galvanostatic cycling of the same units with three different charge and discharge current densities. The current densities were (d) 0.8 A/g, (e) 1.6 A/g and (f) 2.4 A/g. The result indicated that the charging curves of units B to D were more bent and had a higher resistive drop than of unit A, which can be linked to the bending distance in the CV curves. Figure 4f clearly shows a bad charge propagation for unit C and D, which could be related to a poor contact due to the large cavities shown in Fig. 2d. An ideal SC during constant-current charge and discharge exhibits linear charge and discharge curves. The difference in discharge times corresponds to the unit capacitance for the given discharge current. The resistive voltage drop is caused by the ESR of the device.

### Material cost comparison

Aqueous electrolytes such as 1 M sodium sulfate (used here) are much cheaper than common organic electrolytes such as 1 M tetraethylammonium. According to the low-volume price lists from both Alfa Aesar and Sigma-Aldrich, the relative cost, RC, of the organic electrolyte is <30 compared with an RC = 1 for aqueous electrolytes. The AC used was a low-cost product normally used for water purification. The specific surface area was 1200 m^2^/g, and RC = 2 relative to RC = 1 for high-purity flake graphite. ACs designed for SCs with specific surface areas of 1000 to 2500 m^2^/g, are in the RC range of from 20 to 100. The thermally expanded graphite obtained from Superior Graphite was an experimental product; however, in general, expanded graphite has an RC range from 2 to 8 depending on its quality. The RCs are based on quotes from a number of manufacturers to estimate the material cost for pilot-scale production of SCs. The Sigraflex graphite foil current collector has RC = 5 compared with RC = 1 for Aluminum foil. Other materials suited for aqueous electrolytes are stainless steel foil (RC = 30), nickel foil (RC = 50) and titanium foil (RC = 180). The relative costs of current collectors are approximations based on a price comparison in Gheytani *et al*.^9^ and on quotes for Sigraflex graphite foil. All foils had a thickness of 0.2 mm and the relative cost refers to the foil price per unit area.

## Conclusion

The design and processing of a metal-free SC with an aqueous electrolyte was studied and reported. The use of graphite foil as a low-cost and metal-free current collector showed promising results. The carbon-carbon interface between the current collector and electrode resulted in low interfacial resistance and a significant decrease in electrical resistivity, despite large cavities in the electrode material. The ESR was fairly low for an uncompressed aqueous SC, but still large for high power applications. Calendering and cell compression could be needed to lower the ESR further. The low-cost SC concept had approximately half the specific capacitance of commercial SCs but more than 90% lower cost, thus making this approach interesting for large-scale applications without a strict space limitation, such as stationary use.

## Methods

The electrode materials used in this experiment were nanographite for good electrical conductivity and AC for improved surface area and, thus, improved capacitance. NFC was used as a binder to improve strength and stability in the electrodes, as described by Andres *et al*.^16^. The nanographite was processed according to the method described by Blomquist *et al*.^10^ using thermally expanded graphite (SO#5-44-04) from Superior Graphite (Chicago, USA) as the initial material. The nanographite had a specific surface area of 20 m^2^/g. The AC (Pulsorb 208CP) from Chemviron Carbon (Gothenburg, Sweden) had a stated specific surface area of 1200 m^2^/g and was used as-received. The NFC used as a binder was TEMPO-oxidized kraft-pulp NFC prepared according to the method described by Saito *et al*.^17^. Sigraflex graphite foil (F02012TH) from SGL Group (Meitingen, Germany) was used as a current collector.

### Electrode fabrication

Five different samples (Samples A to E) was prepared with a proportion of AC ranging from 50% to 90%. A mixture of nanographite and AC with a combined solid weight of 36 g was dispersed in 800 ml of water for 10 min with an IKA T25 Ultra Turrax (s25 N–25 F dispersion element) at 12 kRPM. NFC was added to a concentration of 10 wt% and the content of solids solids was adjusted to 4% with water. To obtain a well-dispersed suspension, the sample was further dispersed for 20 min, with a IKA T50 Ultra Turrax (S50 N–G45 F dispersion element), at 6 kRPM. This procedure was repeated for all five samples, where sample A) had an active material ratio of 50% AC and 50% nanographite, and samples B, C, D and E had ratios of 60%/40%, C) 70%/30%, D) 80%/20%, and E) 90%/10%, respectively. The binder was excluded from the ratio.

**Figure 5:**
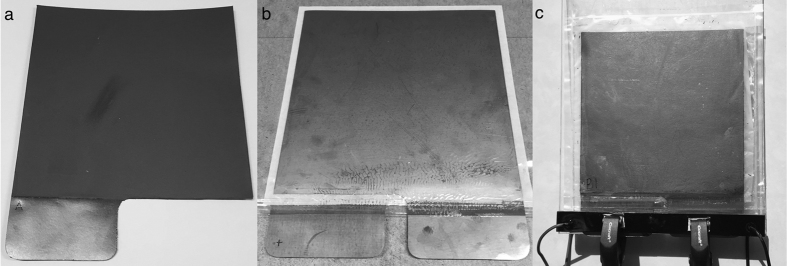
(**a**) The 200 mm × 200 mm electrode-coated graphite foil with a 90 mm × 50 mm contact, (**b**) the assembled SC pouch cell device with two electrode-coated graphite foils and a grease proof paper separator between them, (**c**) electrolyte-filled SC pouch cell device connected to a four-point-probe contact.

The graphite foil current collector was cleaned with 0.5 M sodium hydroxide and rinsed with water. Stainless steel frames of 2 mm thickness were placed on top of the graphite foil. The samples were poured into the frames and leveled with a coating blade to a wet coating thickness of 2 mm. Six frames (three sets of electrodes) per sample were coated and dried at room temperature for 24 h. The electrode-coated graphite foil was cut to an electrode size of 200 mm × 200 mm with a 90 mm-wide and 50 mm-long contact (see Fig. 5a). The electrode-coated graphite foil was further dried in an oven at 105 °C for 90 minutes, and this was followed by weight and thickness measurements. The foil weight was subtracted. The thickness was measured using a Mahr Millitast 1083. The median of five measurements was calculated. This process was repeated in order to obtain two additional electrode-coated graphite foils for each sample, which then were cut to an electrode size of 100 mm × 200 mm to enable higher current densities and scan rates with existing measurement equipment.

The SCs were assembled with two electrode-coated graphite foils, with the electrodes facing each other and with a 210 mm × 210 mm (110 mm × 210 mm for the smaller electrodes) untreated grease proof paper between them. The devices were placed in plastic bags (pouch cell) welded at three edges and then filled with 1 M sodium sulfate electrolyte. The devices remained in electrolyte for 3 h to wet the electrodes and separators. The excess electrolyte was then removed, and the remaining open edge of the plastic bags was welded. A picture of an assembled SC pouch cell device is presented in Fig. 5b. The SC pouch cells were connected to four 90 mm × 20 mm copper contacts with wires for four-point-probe electrochemical measurements (see Fig. 5c).

Furthermore, images of the electrode-coated graphite foils were obtained using a Zeiss Merlin field emission scanning electron microscope (FESEM). Secondary electron images (SEIs) were generated using a 5 kV accelerating voltage and an in-lens detector.

Electrode films were prepared by filtering to allow comparisons of sheet resistance and electrical resistivity between the electrode material itself and the electrode-coated current collector. Ten grams each of samples A to E was filtered through a Millapore Durapore membrane filter (filter type: 0.22 *μ*m GV) and dried at room temperature for 48 h. The electrode films were further dried in an oven at 105 °C for 90 minutes and this was followed by thickness measurements using the same procedure as that for the electrode-coated graphite foils.

### Electrical measurements

Three different electrical measurements were performed on the SC pouch cells; galvanostatic cycling (GC), cyclic voltammetry (CV) and sheet resistance. Sheet resistance measurements were also performed on the filtered electrode films.

Galvanostatic cycling (constant-current charge-discharge) cycles were measured using a LabVIEW-based PXI system. The collected data were analyzed according to the method described by Stoller and Ruoff^11^. The capacitance, *C*, of the SCs was calculated from the discharge curves by using Equation (1):


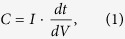


where *I* is the discharge current, *t* is the discharge time and *V* is the cell voltage. The discharge current *I* was set to 1 A **f**or the large cells, resulting in a current density of 0.8 A/g and a discharge time of less than 1 min. The smaller SC units, 100 mm × 200 mm, were cycled at three different current densities (0.8 A/g, 1.6 A/g and 2.4 A/g) in order to measure over a wider range of current loads. The SCs were cycled for 100 cycles at each current density, and the capacitance of the 100th cycle was calculated for each unit to compare the performance of the SCs. Further cycling in 24 h were performed on the smaller units, with a current density of 0.8 A/g to analyze the cycle stability (cyclability). The specific capacitance, *Csp* was calculated by using Equation (2):


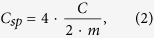


where *m* is the mass of active material in one electrode. The ESR was calculated by dividing the resistive voltage drop, generated between charging and discharging, with the change in current.

Using the same cell configuration and contacts, CV was performed on the smaller units immediately after GC with a Versastat4 and scan rates of 10 mV/s, 20 mV/s and 30 mV/s. The specific capacitance, *Csp*, was calculated from the current plateaus in the discharge curves using equations (1) and (2), and the mean value of three cycles was determined. The current density (A/g) was calculated in the same manner as the specific capacitance (F/g).

The sheet resistance of both the electrode-coated foils and electrode films was measured using a Keithley 2611A four-point-probe system. The electrical resistivity was calculated by multiplying the sheet resistance with the thickness of the electrode.

## Additional Information

**How to cite this article:** Blomquist, N. *et al*. Metal-free supercapacitor with aqueous electrolyte and low-cost carbon materials. *Sci. Rep.* **7**, 39836; doi: 10.1038/srep39836 (2017).

**Publisher's note:** Springer Nature remains neutral with regard to jurisdictional claims in published maps and institutional affiliations.

## Figures and Tables

**Table 1:** Active material composition in sample A to E.

Sample	Active material composition	
	Nanographite	Activated carbon
A	50 *wt*%	50 *wt*%
B	40 *wt*%	60 *wt*%
C	30 *wt*%	70 *wt*%
D	20 *wt*%	80 *wt*%
E	10 *wt*%	90 *wt*%
